# Orthopedics

**Published:** 1904-01

**Authors:** Prescott Le Breton

**Affiliations:** Buffalo


### PROGRESS IN MEDICAL SCIENCE.

### Orthopedics.

CONDUCTED BY PRESCOTT LE BRETON, M. D., Buffalo.

### A NEW PRINCIPLE OF CURING SEVERE CASES OF CLUB-FOOT.

OGSTON (*British Medical Journal,* June 21, 1902), relying on the fact that ossification of the tarsal bones is completed at a late date, has devised a new operation.

After the application of an Esmarch bandage the tendo Achilles is divided. An incision is then made from in front of the external malleolus to the calcaneo-cuboid joint, dividing ali tissues to the bones. By means of a Volkmann's spoon, the bony center of the astragalers is scraped out leaving the cartilaginous shell, and a small amount of bony tissue between the malleoli. If necessary the cuboid and the anterior end of the oscalcis are also scraped. The foot can then be placed in a corrected position, as it is as soft and pliable as a squeezed orange. The incision is sutured and dressings applied. Blood-clot fills the cavities and bones of correct shape reform. This method is applicable to children up to 6 or 8 years old. The after-treatment is considerably shortened and it is believed that the growth of the foot is not affected.

H. Augustus Wilson, (*American Medicine,* January 31, 1903), in commenting upon this operation, says that both the Phelps' and the Ogston's operations are in marked contrast to the non-cutting methods advocated by Lorenz and must direct attention to the necessity for early correction with the least possible cicatrix, to the avoidance of undue restraint and to the early development of muscles which alone can induce normal functions.

Lauenstein, (*Centralblatt f. Chirurgie,* XXX No. 39, quoted by *The Journal of the Amer. Med. Asso.*) advocates this method of enucleating the bony kernel. He advises an *x*-ray examination before operation to enable the surgeon to decide what bones to attack in each individual case.

THE BEST METHOD OF CORRECTING DEFORMITY AT THE HIP.

GIBNEY (*New York Medical Journal,* July 25, 1903,) regularly adopts Gant's subtrochanteric osteotomy in correcting deformity due to hip disease, deeming it more safe and more uniformly successful than forcible correction. He admits that there have been innumerable cases where brisement force has been employed without any outward symptoms, but on the other hand even in selected cases, the old inflammatory process may reappear and even cause abscess. Everyone must admit that it is exceedingly difficult to determine when a joint is ready for the employment of forcible correction. A certain number of cases relapse after osteotomy, but the same may be said for the other method. It has long been his conviction that the plaster-of-paris splint should be kept on a long time after osteotomy, from three to six months. The osteotomy should be subcutaneous, because the osteotome is just as tractable in the hands of a skilful operator as the tenotom. Although many patients prefer a slight flexion after operation, so that they may sit more comfortably, Gibney is convinced that the limb should be left as straight as possible. The patient then walks a great deal better and is in less danger of developing a painful hip in after years.

---

A NEW METHOD OF CORRECTING FLEXION DEFORMITY AT THE KNEE.

WHITMAN (*New York Medical News,* February 7, 1903,) believes that there is no deformity more difficult of correction than this, because of the backward dislocation complicating the flexion and the frequent contracture of the flexor tendons. Usually in straightening such a joint the end of the femur is the fixed point and the tibia is used as a lever. Whitman has had success from using the femur as the lever and the head of the tibia as the fixed point. The child is placed face downward and the head of the tibia held firmly in position on the table, pillows supporting the rest of the body. Massage and forcible stretching of the hamstring tendons are first employed. A carefully applied plaster-of-paris bandage holds the correction obtained after the manipulations. Very little discomfort follows and backward dislocation is less likely to occur. This method is useful also after the subsidence of acute symptoms of various conditions, such as gonorrheal arthritis or typhoid arthritis. Where it is impossible to straighten the limb by this means osteotomy of the femur above the joint may be done.

---

THE DIAGNOSIS OF HIP DISEASE.

LOVETT (*Interstate Medical Journal,* September, 1902, quoted by *Therapeutic Gazette,* February, 1903,) maintains that many cases likely to be classed as tuberculosis hip disease are not tuberculosis. He followed the course of a large number of cases in which the early diagnosis was hip disease. Forty-five of these eventually proved to be tubercular. A group of twenty-one cases, all of which recovered in less than four months, presented at the outset typical symptoms,—atrophy, night-cries, pain in the knee, muscle spasm and loss of gluteal fold. Some of these must conform to Koenig's synovial type of acute infectious coxitis, others traumatic and rheumatic synovitis, or epiphyseal hyperemia. Fourteen other cases resolved themselves into infantile paralysis, osteomyelitis, coxavara, arthritis deformans and seven questionable nontubercular affections. Of all of the signs given "thickening of the trochanter" proved the most reliable. The *x*-ray was also of great value in early diagnosis.

---

THE TREATMENT OF ACETABULAR DISEASE OF THE HIP JOINT.

BRADFORD (*Annals of Surgery,* 1902,) describes an original method of dealing with a tubercular focus confined principally to the acetabulum. The treatment offers special difficulties as drainage of sinuses ordinarily is incomplete and pelvic abscesses are likely to form. The usual excision of the joint does not allow free drainage, as the remainder of the femur interferes. Excision of the acetabulum is too severe. Nature attempts to heal such cases often by subluxating the head of the bone by muscular spasm in the effort to relieve pressure on diseased areas.

Bradford in three successful cases has cut down on the joint posteriorly, dislocated the bone upward and inserted into the acetabulum a glass drainage tube about one inch in diameter, through which applications of carbolic acid and alcohol could be made. The leg is flexed and abducted and held in plaster-of-paris. The tube is kept in place a long time until healthy granulations appear. The patient is allowed to go about on crutches. After healing is complete subtrochanteric osteotomy corrects the position of the leg. This is a life-saving measure introduced to replace amputation at the hip.

---

PRIMARY TUBERCULOSIS OF THE VERTEBRAL ARCHES AND PROCESSES.

EASTMAN (*Amer. Journal of Surgery and Gynecology,* April, 1903,) discusses cases in which the focus is not located in the bodies of the vertebræ. This superficial form is more frequent in adults and no kyphosis follows. Local tenderness exists and patients may continue to work until sinuses appear. Operation and cure are the rule, the scraping of the spinous processes or lanunæ being easy and satisfactory. Of course, this "posterior vertebral disease" is a rare condition.

